# Integration of proteomic data with genome‐scale metabolic models: A methodological overview

**DOI:** 10.1002/pro.5150

**Published:** 2024-09-14

**Authors:** Farid Zare, Ronan M. T. Fleming

**Affiliations:** ^1^ School of Medicine, University of Galway Galway Ireland

**Keywords:** constraint‐based modeling, genome‐scale model, proteomics, systems biology

## Abstract

The integration of proteomics data with constraint‐based reconstruction and analysis (COBRA) models plays a pivotal role in understanding the relationship between genotype and phenotype and bridges the gap between genome‐level phenomena and functional adaptations. Integrating a generic genome‐scale model with information on proteins enables generation of a context‐specific metabolic model which improves the accuracy of model prediction. This review explores methodologies for incorporating proteomics data into genome‐scale models. Available methods are grouped into four distinct categories based on their approach to integrate proteomics data and their depth of modeling. Within each category section various methods are introduced in chronological order of publication demonstrating the progress of this field. Furthermore, challenges and potential solutions to further progress are outlined, including the limited availability of appropriate in vitro data, experimental enzyme turnover rates, and the trade‐off between model accuracy, computational tractability, and data scarcity. In conclusion, methods employing simpler approaches demand fewer kinetic and omics data, consequently leading to a less complex mathematical problem and reduced computational expenses. On the other hand, approaches that delve deeper into cellular mechanisms and aim to create detailed mathematical models necessitate more extensive kinetic and omics data, resulting in a more complex and computationally demanding problem. However, in some cases, this increased cost can be justified by the potential for more precise predictions.

## 1 INTRODUCTION

In the field of systems biology, the integration of omics data with genome‐scale models enhances our understanding of cellular mechanisms and our ability to predict their behavior. Among these omics datasets, proteomics plays a pivotal role in understanding and predicting cellular translational mechanisms. Integrating proteomics data with genome‐scale models enables researchers to bridge between genotype and phenotype, which improves our understanding of the cellular governing machinery. In the past 20 years, several approaches have been developed to leverage proteomics data with genome‐scale models, each with its own strengths and limitations. This review aims to explore and evaluate the diverse methods employed for this integration, highlighting the underlying mathematical principles and detailed methodologies utilized by these approaches. By comprehensively examining these methods, we aim to facilitate an overview of progression in the field and provide insights into their potential applications and challenges. Based on each method's approach for connecting proteomics data with flux values of reactions in a metabolic network, this article categorizes these methods into four distinct groups. Furthermore, we discuss the most important challenges in this area, including the scarcity of in vivo data, the challenges associated with retrieving or calculating enzyme turnover numbers, and the computational complexity required to achieve accurate results. Finally, we summarize the findings of this review and provide suggestions for future studies. By comprehensively and critically investigating each approach, we gain insights into research gaps in this field and potential areas for improvement.

## 2 METHOD SELECTION AND CATEGORIZATION

There are different approaches for leveraging biochemical data in genome‐scale models. These methods can vary from constraining the solution space of metabolic models to developing mechanistic equations and mathematically modeling all transcription and translational processes in detail. Categorizing certain methods can be challenging at times, as they may combine various approaches. Here, we categorize these methods into four distinct approaches of (i) proteomics‐driven flux constraints, (ii) proteomics‐enriched stoichiometric matrix expansion, (iii) proteomics‐driven flux estimation, and (iv) fine‐grained methods. These approaches are explored in greater detail in the following sections. Also, a brief summary of each method, the investigated organism and phenotype, and the type of mathematical problem they generate is provided in Table 1.

**TABLE 1:** Methods for integration of proteomics with genome‐scale models.

Method (year)	Organism (model)	Investigated phenotype	Problem type	Reference
FBAwMC (2007)	*E. coli* MG1655	Growth on single carbon sources growth on mixed carbon media	LP	Beg et al. (2007)
IOMA (2010)	*E. coli* (*i*AF1260)	Metabolic flux distribution	QP	Yizhak et al. (2010)
MADE (2011)	*Saccharomyces cerevisiae* (*i*ND750)	Transition from fermentative to glycerol‐based respiration	MILP	Jensen and Papin (2011)
RBA (2011)	*Bacillus subtilis* *E. coli*	Reasons for limitation of growth rate	LP	Goelzer and Fromion (2011)
MOMENT (2012)	*E. coli* (*i*AF1260)	Growth rate prediction	LP	Adadi et al. (2012)
INIT (2012)	Human metabolic model (iHuman1512)	Tissue‐specific model	MILP	Agren et al. (2012)
CAFBA (2016)	*E. coli* (iJR904)	Overflow metabolism	LP	Mori et al. (2016)
SIMMER (2016)	*Saccharomyces cerevisiae*	Nutrient‐limited yeast	QP	Hackett et al. (2016)
GECKO (2017)	*Saccharomyces cerevisiae* (Yeast7)	Overflow metabolism	LP	Sánchez et al. (2017)
LBFBA (2018)	*Saccharomyces cerevisiae* (*i*MM904) *E. coli* (*i*JO1366)	Flux prediction for carbon source cultures	LP	Tian and Reed (2018)
sMOMENT (2020)	*E. coli* (*i*JO1366)	Overflow metabolism	QP	Bekiaris and Klamt (2020)
ETFL (2020)	*E. coli* (*i*JO1366)	Prediction of feasible mRNA, enzyme concentrations and gene essentiality	MILP	Salvy and Hatzimanikatis (2020)
PAM (2021)	*E. coli* (*i*ML1515)	Wild‐type phenotype	LP	Alter et al. (2021)
XomicsToModel (2021)	Human metabolic model (Recon3D)	Dopaminergic neuronal metabolism	DCA‐LP	Preciat, Wegrzyn, et al. (2021)
GECKO 2.0 (2022)	*Yarrowia lipolytica* (*i*Yali4) *E. coli* (*i*ML1515) *H. sapiens* (Human1) *Kluyveromyces marxianus* (*i*SM996)	Metabolic flux distribution and enzyme usage	LP	Domenzain et al. (2022)

### 2.1 Proteomics‐driven flux constraints

A category of methods termed proteomics‐driven flux constraints involves constraining flux values based on proteomics data. The implementation of this approach can be achieved through three main strategies. The first strategy involves knocking down reactions for which there is no evidence of their corresponding encoded proteins in translational data. The second, more intricate strategy aims to restrict the range of permissible flux values for each reaction. This can be accomplished by constraining reaction flux based on the abundance of their associated enzymes, utilizing mathematical equations such as the Michaelis–Menten equation. The third approach represents the limitation on enzymes available in a cell due to limited accessible cellular volume and limitations on macromolecular concentrations. This type of constraint may apply on total enzyme concentration or limit each enzyme individually. Here, we present methodologies that employ proteomics‐driven flux constraints to enhance the accuracy of flux prediction.

#### 2.1.1 Flux balance analysis with molecular crowding

The integration of quantitative proteomics with genome‐scale models was pioneered with flux balance analysis (FBA) (Orth et al., 2010) with molecular crowding (FBAwMC) in 2007 (Beg et al., 2007). This study discusses how high intracellular concentration of macromolecules such as enzymes affects not only the cell physiology but its functionality. This hypothesis is a motivation to investigate how much our predictions can benefit from considering quantified physical and spatial constraints in the genome‐scale models. This constraint would be mathematically expressed as follows:

(1) $$\sum_{i=1}^{N}v_{i}n_{i}\leq V,$$

where $v_{i}$ and $n_{i}$ represent molecular volume and number of moles of the *i*th enzyme respectively. Also, V represents the maximum feasible volume in a cell that can be occupied by enzymes. To leverage enzyme concentration, Equation (1) is divided by cell mass M which results in

(2) $$\sum_{i=1}^{N}v_{i}E_{i}\leq \frac{1}{C},$$

where $E_{i}=n/M$ represents moles per unit mass concentration for enzyme i and $C=M/V\left[g/\mathrm{mL}\right]$ is the cell density. Limiting concentration of enzymes in a cell further constrains the allowable flux values in a genome‐scale model. To this aim, FBAwMC assumes a proportional relationship between enzyme concentration and metabolic flux $\left(f_{i}=b_{i}E_{i}\right)$, assuming that enzymes operate near saturation. Here,  *i* represents the reaction identifier, with $f_{i}$ and $E_{i}$ denoting the flux value and enzyme concentration corresponding to the *i*th reaction, respectively. The coefficient $b_{i}$ is a variable of reaction mechanism, kinetic parameters, and metabolic concentrations. FBAwMC method leverages enzyme constraint in Equation (2) into metabolic flux formulation, which results in a constraint on metabolic fluxes in the reaction as follows:

(3) $$\sum_{i=1}^{N}a_{i}f_{i}\leq 1.$$

In this equation, $a_{i}={\mathrm{Cv}}_{i}/b_{i}$, where $a_{i}$ is crowding coefficient corresponding to reaction *i*. $a_{i}$ measures how much an individual reaction contributes to the total occupancy of the cell's volume by enzymes, taking into account both the size of the enzymes and their activity levels. By minimizing mean square deviation between measured and predicted maximal growth rate, FBAwMC calculates an average crowding coefficient $\bar{a}$. This coefficient can be a variable of cell type or substrate. Several subsequent methods, including MOMENT (Adadi et al., 2012), have built upon the assumptions of FBAwMC (Chang et al., 2021).

#### 2.1.2 Metabolic adjustment by differential expression

Metabolic adjustment by differential expression (MADE) is a method that maps expression data from different conditions onto a metabolic network model without relying on arbitrary expression thresholds. MADE utilizes the statistical significance of changes in gene or protein expression to establish a set of constraints that are applied to adjust the metabolic network model. By utilizing two or more condition‐specific gene/protein expression data, MADE determines the expression changes between different conditions. Through the implementation of Boolean rules and statistical parameters such as *p*‐values, MADE generates a binary representation of expression data. The primary goal of MADE is to identify a sequence of binary expression states while minimizing discrepancies between consecutive states and their corresponding differences in mean expression levels. MADE achieves this by maximizing the sum of objective function values across all conditions, adhering to a regulated FBA formalism that encompasses a standard FBA, Boolean rules, and a minimum value for the objective flux to ensure a viable flux distribution. Ultimately, this problem translates into a mixed integer linear programming (MILP) problem (Jensen & Papin, 2011).

#### 2.1.3 Metabolic modeling with enzyme kinetics

Metabolic modeling with enzyme kinetics (MOMENT) considers maximum cellular capacity for proteins (limitation of total available enzymatic pool). As a result, MOMENT requires the fraction of total protein that is devoted to metabolic enzymes as the first input in order to apply it as one of the constraints to the model. Secondly, MOMENT, considers each enzymatic reaction flux to have a value less than or equal to the product of the corresponding enzyme(s) concentration and turnover rate. This product is used to modify the upper bound on reaction fluxes and further constraints the model. MOMENT also accounts for isozymes, protein complexes and functional enzymes using Boolean rules. It retrieves enzyme turnover numbers from databases such as BRENDA and the system for the analysis of biochemical pathways‐reaction kinetics (SABIO‐RK) database (Chang et al., 2021; Wittig et al., 2006). Furthermore, for those enzymes with missing turnover number, the average turnover number from other species is considered. MOMENT proposes a correlation between enzyme turnover rates, molecular weights, and flux rates. It assumes that higher enzyme turnover rate or molecular weight results in higher fluxes in associating enzymatic reactions (Adadi et al., 2012).

#### 2.1.4 Integrative network inference for tissues

Integrative network inference for tissues (INIT) algorithm leverages gene expression data from BioGPS (Agren et al., 2012; Wu et al., 2009), and publicly available datasets; Human Body Index Transcriptional Profiling (GSE7307), metabolomic data from Human Metabolome Database (HMDB) (Wishart et al., 2007), and proteomics data from Human Protein Atlas (HPA) (Berglund et al., 2008; Uhlén et al., 2005, 2010), to exclude reactions that there is no evidence of their associated protein or gene, and allocate greater flux to reactions with higher enzyme concentrations, thus making a tissue‐specific model. Considering human cells secreting a much broader spectrum compounds into the blood than the microbial cells secret into their extracellular space, INIT, relaxes the steady state constraint for metabolites in the model. Thus enables metabolites with nonzero net flux to either be consumed in the biomass reaction or secreted from the cell. In order to define an objective function, INIT uses a weighted objective function based on gene expression and proteomics data. In cases where the weights are defined based on the proteomics data, they can be assigned to values of 10, 15, and 20, for low to high concentration of proteins, and negative values when the corresponding protein is absent. On the other hand, where the data regarding a reaction comes from gene expression level, weights would be calculated based on the following formula:

(4) $$w_{i,j}=5\log\left(\frac{{\text{signal}}_{i,j}}{{\text{average}}_{i}}\right),$$

where $w_{i,j}$, weight of gene i in tissue j is calculated by dividing signal of gene i in tissue j by the average signal across all tissues. By maximizing the sum of all reaction weights in the model, the INIT algorithm prioritizes reactions with higher gene expression or proteomics values to ensure that the network accurately reflects the metabolic activities most likely to occur in the specific tissue. Subsequently, the problem is solved using MILP (Agren et al., 2012).

#### 2.1.5 Linear bound FBA

Linear bound FBA (LBFBA) establishes novel upper and lower bounds for each reaction based on the expression levels of the reaction. The flux bounds assume a linear correlation with gene/protein expression data. These expression levels are determined by applying gene‐protein‐reaction (GPR) rules to expression data, along with constant parameters derived from independent training sets. LBFBA employs training set data to determine the parameters that correlate expression data to flux bounds and utilizes another independent dataset to evaluate the accuracy of the algorithm. The determination of these parameters in LBFBA is achieved through linear programming. Similar to parsimonious flux balance analysis (pFBA) (Lewis et al., 2010), LBFBA minimizes the absolute values of all fluxes while considering the smallest total flux bound violations of the expression‐based constraints as its objective function. The advantage of LBFBA over other similar approaches lies in its ability to establish reaction‐specific bounds (Tian & Reed, 2018).

#### 2.1.6 XomicsToModel

XomicsToModel is a semi‐automated pipeline for constructing specific genome‐scale metabolic models from a single generic reconstruction using omics data or by applying context‐specific constraints (Preciat, Wegrzyn, et al., 2021). In comparison with previous model extraction methods, such as GIMME (Becker & Palsson, 2008), iMAT (Zur et al., 2010), MBA (Jerby et al., 2010), and mCADRE (Wang et al., 2012), which were designed to build flux consistent context‐specific models, a significant advantage of the XomicsToModel pipeline is its ability to extract a context‐specific model that is stoichiometrically consistent, flux consistent, and thermodynamically consistent.

The XomicsToModel pipeline is capable of integrating various types of data, including all combinations of transcriptomic, proteomic, metabolomic, and bibliomic data retrieved from the literature. It can also utilize all of the aforementioned data types in qualitative, semi‐quantitative, and quantitative formats. The flexibility to integrate multiple complementary datasets enables context‐specific models to make more accurate predictions (Opdam et al., 2017). This feature of the XomicsToModel pipeline enhances its predictive accuracy and makes it an efficient tool for various case studies with diverse sets of available data.

Similar to other methods in the proteomics‐driven flux constraints category, the XomicsToModel pipeline constrains metabolic fluxes by determining active genes using transcriptomics, proteomics, and bibliomics data. From bibliomic data, the activity of each gene is determined based on previous literature. Likewise, using proteomics data, a gene is considered active if its associated protein surpasses a specific threshold level. Finally, transcriptomic data provide quantitative gene expression levels, allowing this pipeline to determine the activity of each gene based on a specific threshold. In the end, the integrated gene activity results from bibliomic, proteomic, and transcriptomic data lead to the identification of each reaction's activity based on gene‐protein‐reaction associations. In cases of conflicts between omics data, the default setting is that bibliomic data takes priority over others.

To create a thermodynamically consistent COBRA model, the XomicsToModel pipeline considers that the flux distribution in a subset of the metabolic network should adhere to thermodynamic laws. According to the second law of thermodynamics, each positive reaction flux in the network must correspond to a negative net change in chemical potential. Since reaction fluxes and directions in a COBRA model are specified within each reaction's defined bounds, thermodynamically inconsistent flux bounds can lead to thermodynamically infeasible flux distributions. In a relative study (Desouki et al., 2015), it is revealed that minimizing the one‐norm of flux values in metabolic networks results in a thermodynamically feasible flux distribution. The XomicsToModel pipeline trades‐off one‐norm minimization for thermodynamic feasibility with cardinality optimization to optimize for or against inclusion of different sets of reactions in proportion to weights on each reaction (Fleming et al., 2023).

The XomicsToModel pipeline is a 21‐step procedure that also encompasses tasks such as data preprocessing, model modifications, identification of flux and thermodynamically consistent subsets, and model extraction. This comprehensive procedure is implemented in MATLAB and is available as a function in the COBRA toolbox (Heirendt et al., 2019). The XomicsToModel pipeline offers two model extraction algorithms: the thermoKernel algorithm, which extracts a minimal thermodynamically flux‐consistent model (Preciat, Moreno, et al., 2021), and FASTCORE (Vlassis et al., 2014), which is capable of extracting a minimal flux‐consistent model. As a case study, this literature extracts a context‐specific genome‐scale metabolic model of dopaminergic neuronal metabolism (Preciat, Moreno, et al., 2021) from Recon3D, a generic human metabolic reconstruction (Brunk et al., 2018).

### 2.2 Proteomics‐enriched stoichiometric matrix expansion

It is possible to utilize sophisticated mathematical approaches to incorporate proteomics data into genome‐scale models, but is crucial to also maintain computational tractability of the corresponding optimization problem(s). Addition of linear inequalities to a standard FBA problem (Orth et al., 2010). enables the utilization of linear optimization to solve the system. In this paper, we highlight several key methodologies that leverage proteomics data in genome‐scale models through the expansion of the stoichiometric matrix.

#### 2.2.1 Genome‐scale model with enzymatic constraints using kinetic and omics data (GECKO 1.0, 2.0, and 3.0)

Genome‐scale model with enzymatic constraints using kinetic and omics data (GECKO) is a methodology that incorporates enzymatic reactions, leveraging quantitative proteomic data (Sánchez et al., 2017). By applying enzymatic constraints to a genome‐scale metabolic model of *Saccharomyces Cerevisiae* (Yeast7) (Aung et al., 2013), GECKO generates an enzyme‐constrained metabolic model named ecYeast7. This approach aims to narrow down the feasible solution space and claims to reduce flux variability for a significant portion (60%) of metabolic reactions. Turnover numbers for enzymes are obtained from the BRENDA database (Schomburg et al., 2004). In the GECKO approach, enzymes are considered as pseudo‐substrates in reactions, resulting in proteomics constraints being placed on their utilization.

To integrate quantitative proteomics data with the genome‐scale model, new exchange reactions are added to the model to allow the enzymes enter the system, and the upper bound of these exchange reactions would be the enzyme abundance. A simple example is illustrated below in Equations ((5), (6), (7)).

(5) $$R_{e_{i}}:\;\to e_{i},$$

(6) $$R_{j}:\frac{1}{k_{\mathrm{cat}}^{i,j}}e_{i}+\mathrm{aA}+\mathrm{bB}\to \mathrm{cC}+\mathrm{dD},$$

while

(7) $$0\leq V_{e_{i}}\leq \left[E_{i}\right].$$

Thus after applying the mass balance on enzyme *i*, we have

(8) $$-\frac{1}{k_{\mathrm{cat}}^{i,j}}\times V_{j}+V_{e_{i}}=0,$$

(9) $$V_{j}\leq k_{\mathrm{cat}}^{i,j}\times \left[E_{i}\right].$$

**FIGURE 1:**
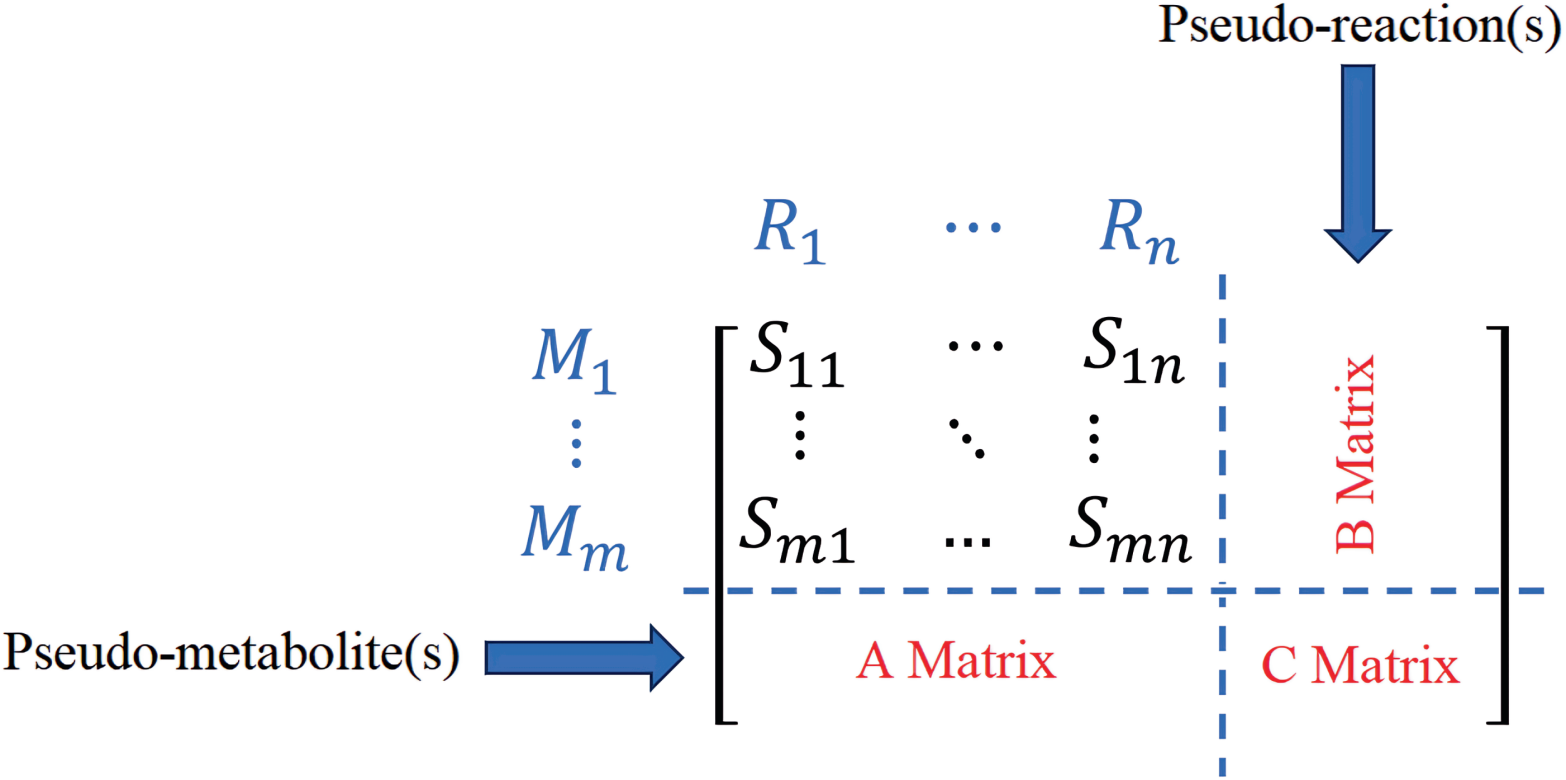
The schematic depicts the inclusion of enzymatic constraints into the stoichiometric matrix. The definitions of the A, B, and C matrices differ between the GECKO and sMOMENT methods. In GECKO 1 and 2, the A matrix contains $-1/k_{\mathrm{cat}}$ for each enzyme, the B matrix is a matrix of zeros, and the C matrix is an identity matrix, whereas in GECKO 3.0, the molecular weights are leveraged into the stoichiometric coefficients, resulting in the A matrix containing $-{\mathrm{MW}}_{j}/k_{{\mathrm{cat}}_{j}}$ values for each enzyme. In sMOMENT, only one pseudo‐metabolite and one pseudo‐reaction are added to the stoichiometric matrix for limiting available protein pool to the cell. As a result, the A matrix becomes a horizontal vector of $-{\mathrm{MW}}_{j}/k_{{\mathrm{cat}}_{j}}$, the B matrix is a vertical vector of zeros, and the C matrix is a single integer.

Here $R_{e_{i}}$ represents the pseudo‐reaction that is added to the stoichiometric matrix to account for enzyme abundance, and $e_{i}$ represent the enzyme as a reactant. $R_{j}$ represent the associated reaction which considers the enzyme as one of the reactants. Finally, Equation (7) constraints the flux of artificial exchange reaction $V_{e_{i}}$ to have any values less or equal to the enzyme abundance $\left[E_{i}\right]$. This is mathematically accomplished by introducing additional rows to the stoichiometric matrix to represent enzymes, and new columns to represent the utilization of each enzyme. Each enzyme's stoichiometric coefficient is set to $-1/k_{\mathrm{cat}}$, in the reactions it catalyzes, and equals one in its associated exchange reaction. A schematic of stoichiometric matrix expansion in different versions of GECKO and a comparable method called short MOMENT (sMOMENT) is shown in Figure 1 (Bekiaris & Klamt, 2020). By expanding the S‐matrix and adding one row for each enzyme, the GECKO method accommodates isozymes and promiscuous enzyme relationships, allowing multiple enzymes to participate in a single reaction and one enzyme to catalyze multiple reactions.

GECKO 2.0 is an update to the original version of GECKO (Domenzain et al., 2022). This updated version expands the method for generating enzyme‐constraint models for more organisms. GECKO 2.0 obtains turnover numbers from BRENDA database from the same organism, or from the phylogenetically closest organism. when data for a desired organism is missing. This is a considerable advantage over its previous version where turnover numbers where retrieved regardless of organism. GECKO 2.0 also introduced a repository that contains enzyme‐constrained models. This *ecModels repository* functions as a continuous integration implementation that serves as a repository for ecModels of relevant organisms. It automatically updates the catalogue of ecModels whenever modifications are detected in the original GEM source repository or the GECKO toolbox. The generated ecModels are stored in a version‐controlled manner in the container repository (https://github.com/SysBioChalmers/ecModels), supporting formats such as SBML and MATLAB files. GECKO 2.0 generated four models which were iYali4, for the oleaginous yeast *Yarrowia lipolytica* (Kerkhoven et al., 2016); iSM996, for thermotolerant yeast *Kluyveromyces marxianus* (Marcišauskas et al., 2019); iML1515, for *E. coli* bacterium (Monk et al., 2017); and Human1, for *H. sapiens* metabolism (Robinson et al., 2020). Recently, a new version of GECKO was released, GECKOpy, which was implemented in the python programming language (Muriel et al., 2023).

GECKO 3.0 is the latest version of GECKO algorithm, which was recently published (Chen et al., 2024). The most significant enhancement of GECKO 3.0 over its previous versions is the use of the deep learning‐based model DLKcat to predict *k*_cat_ values for enzymatic reactions that lack available data in databases (Li et al., 2022). Prediction of kinetic parameters using deep learning models potentially enhances the kinetic modeling of enzymatic reactions and improves predicted reaction rates in genome‐scale models (Li et al., 2022). In the last version, GECKO method applies molecular weight ${\mathrm{MW}}_{i}$ of each enzyme directly into the stoichiometric matrix of metabolic network. As a result, the stoichiometric coefficient of each enzyme becomes $-\mathrm{MW}/k_{\mathrm{cat}}$. Additionally, GECKO 3.0 introduces a light model which eliminates enzyme usage reactions from the model and only considers the protein cost of each enzyme. This approach is similar to sMOMENT but differs in its computational implementation as sMOMENT is Python‐based, whereas the GECKO approach uses MATLAB (Bekiaris & Klamt, 2020).

#### 2.2.2 Short metabolic modeling with enzyme kinetics

While MOMENT was initially categorized as a proteomics‐driven flux bound method, its updated version, abbreviated as sMOMENT, aims to incorporate protein pool constraints into the stoichiometric matrix, thereby placing it in the “Proteomics‐Enriched Stoichiometric Matrix Expansion” category (Bekiaris & Klamt, 2020). sMOMENT imposes a maximum value for the protein pool available to the cell so that the total metabolic enzyme's mass in the cell should not exceed a threshold *P*:

(10) $$\sum g_{i}\cdot {\mathrm{MW}}_{i}\leq P,$$

where, *g*_*i*_, and ${\mathrm{MW}}_{i}$ represent concentration and molecular weight of enzyme i respectively. Also, the flux value of a reaction catalyzed by the enzyme i is limited by available enzyme in the cell, same as similar approaches using Equation (9), which sMOMENT, retrieves enzyme turnover number $k_{\mathrm{cat}}$, from SABIO‐RK and BRENDA databases. Substituting Equation (9) into Equation (10), introduces a constraint on the protein pool in the cell.

(11) $$-\sum v_{i}\cdot \frac{{\mathrm{MW}}_{i}}{k_{\mathrm{cat},i}}+v_{\text{pool}}=0;\;v_{\text{pool}}\leq P.$$

This constraint is incorporated by adding a pseudo‐metabolite and a pseudo‐reaction to the stoichiometric matrix where every element of the pseudo‐metabolite's row equals the molecular weight divided by the turnover rate ${\mathrm{MW}}_{i}/k_{\mathrm{cat},i}$ of the enzyme associated with each reaction, and the upper bound of the pseudo‐reaction is set to *P*. Figure 1 shows how this constraint is added to the stoichiometric matrix. As in this approach, each reaction is considered to be catalyzed by one enzyme, sMOMENT selects the enzyme with the minimum enzyme cost *c*_*i*_, where $c_{i}={\mathrm{MW}}_{i}/k_{\mathrm{cat},i}$ in the case of isozymes.

To integrate enzyme usage explicitly, sMOMENT sets an upper bound on flux values catalyzed by the enzyme as follows:

(12) $$\sum_{i\in R\left(E^{i}\right)}\frac{v_{i}}{k_{\mathrm{cat},i}}\leq \left[E^{i}\right],$$

where, $\left[E^{i}\right]$ represents concentration of enzyme *i*, and $R\left(E^{i}\right)$ denotes the set of reactions catalyzed by enzyme *E* ^*i*^. sMOMENT adds a pseudo‐metabolite and a pseudo‐reaction to apply this relationship into the genome‐scale model. In conclusion, sMOMENT is able to integrate total metabolic enzyme concentration with genome‐scale models where, by adding only one metabolite and reaction to the model makes an efficient model from a computational aspect. On the other hand, sMOMENT is able to leverage explicit enzyme concentration; however, for each of these concentrations, one extra row and column is added to the stoichiometric matrix which in the case where all the enzyme concentrations are available, the sMOMENT model would have the same size as a GECKO model. Hence, sMOMENT would be a proper approach where non or limited explicit enzyme concentration is available.

The Automatic integration of Protein Allocation Constraints in Metabolic Networks (AutoPACMEN) toolbox implements the sMOMENT method. AutoPACMEN utilizes a Systems Biology Markup Language (SBML) version of metabolic models as input to generate sMOMENT models by extending their stoichiometric matrix. Second, it calibrates the sMOMENT model parameters using in vivo data. AutoPACMEN is openly accessible at https://github.com/ARB-Lab/autopacmen.

#### 2.2.3 Protein allocation model

Protein allocation model (PAM) classifies proteins into four distinct groups: (i) translational proteins, which include ribosomes, (ii) metabolically active enzymes, (iii) un‐utilized enzymes, and (iv) housekeeping proteins that maintain a consistent abundance regardless of environmental conditions and do not contribute to condition‐specific phenotypes (Alter et al., 2021). PAM imposes a total constraint on the protein concentration as follows:

(13) $$\phi_{P,c}=\phi_{T}+\phi_{\mathrm{AE}}+\phi_{\mathrm{UE}},$$

where, $\phi_{P,c}$ is the constant total protein mass concentration and $\phi_{T}$, $\phi_{\mathrm{AE}}$, and $\phi_{\mathrm{UE}}$ are the variable protein mass fractions of the translational protein, active enzymes, and unused enzyme sector, respectively. Using experimental data, PAM method assigns $\phi_{P,c}$ to 81% of the measured protein mass which equals $0.26\;g/g_{\mathrm{cdw}}$. PAM sets the protein mass fraction of the translational protein sector as a linear function of the growth rate. The parameters in this linear function are calculated for *E. coli* by fitting a linear equation to measured, cross‐conditional concentration data of the translational protein sector. The unused enzyme sector is modeled as a negative linear function of the substrate uptake flux to reflect the adaptive allocation of cellular resources based on metabolic demand. The parameters in this linear function are determined from ME‐model simulations of underutilized enzymes (O'Brien et al., 2016). To account for the enzyme demand of metabolic fluxes, the PAM method integrates enzyme mass balances for all relevant metabolic reactions with respect to the GECKO framework. Furthermore, the PAM method assumes the active enzyme sector to be equal to the summation of the concentrations of all enzymes constituting the metabolically active enzyme sector. The PAM method assumptions lead to an extended version of FBA with additional protein allocation constraints.

### 2.3 Proteomics‐driven flux estimation

The third category of approach considers a kinetic mechanism like Michaelis–Menten kinetics, for estimating flux values of enzymatic reactions and bridge between proteomics data and metabolic reaction fluxes. Consequently, kinetic equations enable the calculation of flux values using enzyme and metabolic concentrations and kinetic parameters. Within this category, macromolecules and their associated reactions are not added to the stoichiometric matrix, thereby maintaining the original size of the matrix of the metabolic model (M‐model). Instead, the metabolic flux values are connected to macromolecule concentrations using enzyme kinetic equations. In some methods, due to data deficiency, calculating flux values for all reactions is not practical. In such cases, first, flux values are computed for internal core reactions within the network. Then, using mass balance and the stoichiometric matrix, flux values for the remaining reactions are also determined. Furthermore, in certain situations, it may not be possible to precisely determine flux values for all reactions only using mass balance equations due to the high number of variables in the metabolic network. Consequently, an objective function is employed to narrow down the solution space and assist in obtaining a meaningful answer. One of the most common examples of an objective function is finding flux values that align most consistently with calculated flux values for core reactions. This section introduces and investigates the most significant methods utilizing this approach.

#### 2.3.1 Integrative omics‐metabolic analysis

Integrative omics‐metabolic analysis (IOMA) is one of the pioneers of integrating proteomics with genome‐scale models (Yizhak et al., 2010). In this method, metabolic levels for each reaction are catalogued as substrate and product concentrations. This approach utilizes a Michaelis–Menten‐like kinetic rate equation to calculate flux values from protein abundance data and metabolic levels. These calculations also require product and substrate concentrations, and enzyme dissociation constant $\left(k_{m}\right)$ and enzyme turnover rates for both forward and backward directions ($k_{\mathrm{cat}}^{+}$ and $k_{\mathrm{cat}}^{-}$). Consider that according to data deficiency, IOMA calculates flux values for a limited number of reactions, ideally core reactions with the most impression on the network. After calculating the flux values for a number of core reactions, IOMA conducts a quadratic programming (QP) problem to predict all flux values in a metabolic network. IOMA method aims to find flux distribution that: (i) satisfy stoichiometric mass‐balance and reaction directionality constraints and (ii) be the most consistent with flux values calculated from kinetic equations. IOMA uses an error variable for each core reaction to relax the optimization problem and guarantee a feasible solution for the optimization and aims to minimize this error variable as the objective of the optimization process. This error is defined as follows:

(14) $${\bar{\epsilon }}_{\mathrm{ji}}=\frac{{\bar{v}}_{\mathrm{ji}}\cdot e_{\mathrm{ref},i}}{e_{\mathrm{ji}}}-a_{\mathrm{ji}}^{+}{\bar{v}}_{\max,i}^{+}+a_{\mathrm{ji}}^{-}{\bar{v}}_{\max,i}^{-}.$$

In this equation, ${\bar{\epsilon }}_{\mathrm{ji}}$ is the error term for reaction i under condition j, ${\bar{v}}_{\mathrm{ji}}$ is the average net flux through reaction i under condition j, $e_{\mathrm{ref},i}$ is the enzyme concentration in the reference state, $e_{\mathrm{ji}}$ is the enzyme concentration under condition j, $a_{\mathrm{ji}}^{+}$ and $a_{\mathrm{ji}}^{-}$ are the saturation values for the forward and backward directions respectively, and ${\bar{v}}_{\max,i}^{+}$ and ${\bar{v}}_{\max,i}^{-}$ are the average maximum flux rates for the forward and backward reactions respectively, derived from the product of the enzyme turnover rate and $e_{\mathrm{ref},i}$. IOMA is able to leverage relative proteomic abundance data by applying the reference enzyme concentration.

#### 2.3.2 Constrained allocation flux balance analysis

Proteomic allocation models (PAM) and constrained allocation flux balance analysis (CAFBA) are two comparable methodologies that have investigated the allocation of proteins within cells. CAFBA introduces the concept that bacterial proteomes are distributed among distinct sectors, with this distribution being dependent on cellular growth rate (Mori et al., 2016). Consequently, cells strive to allocate their proteins in a manner that maximizes their growth rate. CAFBA categorizes protein sectors into four parts: (i) ribosome‐associated proteins (R‐sector), (ii) biosynthetic enzymes (E‐sector), (iii) proteins involved in carbon intake and transport (C‐sector), and (iv) core housekeeping proteins (Q‐sector). The first constraint is established by setting the sum of the proteome fractions for each sector equal to unity. The fractions for each sector are explicitly defined; for example, the ribosomal sector is linearly correlated with the growth rate. The carbon catabolic sector is assumed to be linearly correlated with carbon intake flux, while the biosynthesis sector is determined by enzyme, substrate, and product concentrations. By assuming irreversible reactions and enzyme saturation, the biosynthesis sector can be represented as a linear combination of weighted reactions. Since housekeeping proteins are independent of growth, they require no further investigation. Eventually, the flux through enzymatic reactions are calculated using Michaelis–Menten kinetics equations. To linearize the kinetic equation, CAFBA assumes that enzymatic reactions are substrate saturating, meaning that the Michaelis constant of the reaction is negligible compared to the substrate concentration $\left(\left[S\right]\gg K_{M}\right)$, and the non‐linear part of the Michaelis–Menten kinetic formula is simplified to: $\frac{\left[S\right]}{\left[S\right]+K_{M}}\approx 1$. Also, CAFBA avoids absolute flux values which leads to a MILP problem by splitting reversible reactions into forward and backward reactions, thus turning the problem into a LP problem. CAFBA further constrains the genome‐scale model using protein allocation constraints and solves the problem using classic FBA. CAFBA and PAM share several common considerations. However, a notable advantage of PAM over CAFBA is the incorporation of constraints directly into the stoichiometric matrix, which simplifies the problem.

#### 2.3.3 Systematic identification of meaningful metabolic enzyme regulation

Systematic identification of meaningful metabolic enzyme regulation (SIMMER) involves the quantification of metabolite and enzyme concentrations through mass spectrometry, while reaction fluxes are evaluated based on complementary experimental data and the established stoichiometry of the metabolic network (Hackett et al., 2016). Subsequently, the concentrations of enzymes and metabolites are linked to metabolic reaction fluxes by applying Michaelis–Menten kinetics equation to each individual reaction. For each reaction one or more kinetic equations are generated that differ in terms of inclusion of regulators, and the calculated flux values are compared against the measured fluxes to identify the most predictive model. Finally, the most precise mathematical kinetic equation is employed to estimate flux values for each reaction. In this method, enzyme turnover numbers are computed using the non‐negative least squares (NNLS) method.

### 2.4 Fine‐grained methods

Fine‐grained methods are the most comprehensive category. With this strategy, protein expression is meticulously considered, and mechanistic equations are derived for different levels, ranging from mRNA to ribosome expression. Consequently, this approach necessitates a larger amount of biological and kinetic data but yields more accurate predictions. In this section, we present a selection of notable publications within this category.

#### 2.4.1 Metabolism and expression models

Metabolism and expression models (ME‐models) enable integration of proteomics with genome‐scale models. Unlike M‐models, which connect genes to their associated reactions using Boolean rules, ME‐models integrate stoichiometric representations of metabolism and macromolecular expression into a unified model. This approach establishes a mutual dependency between metabolism and macromolecular expression: metabolism relies on macromolecular expression to provide enzymes for catalyzing reactions, while macromolecular expression depends on metabolism for the supply of precursors. This cyclic dependency results in a more comprehensive and accurate representation of cellular systems.

ME‐models explicitly stoichiometrically represent transcriptional, translational, post‐translational and enzymatic turnover, which is either coupled to enzyme turnover constraints (Thiele et al., 2012) or coupled to doubling time (Lerman et al., 2012). This explicit modeling of the cellular machinery network equips ME‐models with the ability to predict protein costs within a model and identify the most efficient way for a cell to allocate resources for growth. Consequently, ME‐models excel at forecasting cellular protein adaptation and allocation under diverse conditions. One of the key distinctions between ME‐models and M‐models lies in their constraint matrices. In addition to stoichiometric constraints and maximization of a biomass reaction, ME‐models incorporate multiple metabolite and enzyme coupling coefficients in the form of linear inequalities, resulting in a linear optimization problem (Thiele et al., 2012). Due to the differences in magnitude of stoichiometric coefficients and differences in magnitude of flux bounds, ME‐models are multi‐scale and are numerically challenging to solve. Novel linear optimization solvers were developed, incorporating a quadruple precision arithmetic were developed (Ma et al., 2017), to enable efficient and reliable solution of such multi‐scale linear optimization problems. In ME‐models, when variable bounds are considered to be a function of growth rate. the result is a quasi‐concave maximization problem, although in certain scenarios one can consider them quasi‐linear (Yang et al., 2016). To solve multi‐scale ME‐model when variable bounds are considered to be a function of growth rate, solveME (Yang et al., 2016), a specialized solver utilizing quadruple precision enables one to reliably solve growth maximization problems within ME‐models. For given ME‐model, quasi‐concave maximization may predict a unique flux vector, in contrast to FBA with a M‐model, where multiple alternate optimal solutions may exist.

The initial step in reconstructing an ME‐model involves integrating metabolic and a macromolecular synthesis machinery networks. These networks are mathematically represented by the metabolic matrix (M‐matrix) and expression matrix (E‐matrix), respectively. Given their interdependence, addressing the overlap between them becomes a mathematical necessity in creating a unified model. One such integration involves incorporating enzymes into their corresponding reactions to establish a direct connection between enzymes and the reactions they catalyze. This step is achieved by reformulating the stoichiometric matrix.

A ME‐model was first reconstructed for *E. coli* (Thiele et al., 2012) by integrating an M‐model of *E. coli* (*i*AF1260) (Feist et al., 2007) with the first genome‐scale stoichiometric network of macromolecular synthesis machinery of *E. coli* (Thiele et al., 2009). The resulting ME‐model represented the function of almost 2000 genes. It extended the network of the M‐model by adding transcription and translation reactions for each metabolic gene in the M‐matrix. The constructed ME‐matrix outperformed the *i*AF1260 model in predicting growth in different environments and correctly predicted knockout phenotypes. Subsequently, a simplification of that approach was then applied to develop an ME‐model for *Thermotoga maritima* (Lerman et al., 2012). A subsequent *E. coli* ME‐model (iOL1650‐ME) was developed that contains 1295 functional protein complexes, accounting for 80%–90% of *E. coli*'s expressed proteome by mass (O'Brien et al., 2013). Subsequently it was enhanced by incorporating protein translocation pathways, categorizing proteins into specific cellular compartments, integrating experimentally determined rates for translocase catalysis and porin diffusion, and introducing a unique membrane constraint to mathematically model cell structure, named iJL1678‐ME (Liu et al., 2014).

To facilitate development of ME‐models, a software package entitled COBRAme (Lloyd et al., 2018) was developed. COBRAme achieves the simplification of ME‐models by revising how explicit coupling constraints for metabolites are implemented. Additionally, it lumps together significant cellular processes, including transcription and translation, into a consolidated ME‐model reaction. COBRAme is encoded in Python and leverages COBRApy (Ebrahim et al., 2013). A platform for modeling with M‐models. The first ME‐model for a gram‐positive bacterium, *Clostridium ljungdahlii*, were reconstructed by extending iHN673 (Nagarajan et al., 2013), an existing M‐model of *C. ljungdahlii*, and constructing a new M‐model named iJL680 (Liu et al., 2019). Subsequently, an E‐Matrix of *C. ljungdahlii* was constructed and integrated into iJL680 metabolic network using COBRAme to create iJL965‐ME, marking the first ME‐model for *C. ljungdahlii*. Additionally, a recent development known as DynamicME, as presented by introduced an algorithm capable of transforming ME‐models into dynamic ME‐models (Yang et al., 2019). This algorithm considers time‐dependent variables, including protein concentration and growth rate, to enhance the predictions. In summary, ME‐models offer the capability to simulate a wide range of phenotypes, extending beyond metabolic reactions. However, ME‐models present computational challenges due to their multi‐scale and high‐dimensional nature.

#### 2.4.2 Resource balance analysis

Resource balance analysis (RBA) focuses on the distribution of proteins between cellular processes (Goelzer et al., 2011; Goelzer & Fromion, 2011). RBA considers three main constraints in the model to further reduce the solution space and enhance the model accuracy: (i) limitation of growth rate caused by cell structure constrains the cell growth rate as a function of ribosome and protein concentrations; (ii) constraining the maximum number of macromolecules in a cell; (iii) constraining the metabolic synthesis of precursor consumed during synthesis of a protein, as a function of growth rate, ribosome and protein concentration. RBA can be said to be an extension of the FBA framework that captures the resource allocation between competing cellular processes. RBA assumes that each metabolic flux correlates with its corresponding enzyme concentration through the following equation:

(15) $$|v_{P}|=k_{P}\times P,$$

where $v_{P}$ represents the metabolic flux associated with the enzyme P, $k_{P}$ denotes the efficiency of the enzyme *P*, and P stands for concentration of enzyme *P*. Since both enzyme efficiency and enzyme concentration are greater than zero, the other side of the equation must also be non‐negative. However, flux values are vectors, not scalar numbers, which means they can have negative components, indicating their direction. To address this issue, the absolute value of the metabolic flux is considered in the Equation (15). The enzyme concentration *P* plays a critical role in further constraining the model, by adding relationships of ribosomes allocation in metabolic networks. This is done by introducing constraints that must be satisfied for accurate predictions of cellular growth and metabolism such as constraints on the total density of the cell with respect to protein and ribosome density, and constraints on the growth rate using translational efficiency and ribosome concentration. The use of absolute values in the constraints introduces discontinuities at zero, creating corners or kinks in the constraint boundaries, thereby making the problem non‐smooth. Accordingly, RBA results in a non‐smooth convex constraint‐based feasibility problem. Non‐smooth (also known as non‐differentiable) functions introduce complexities in the optimization process due to the lack of derivatives, potential multiple optima, and slower convergence, however the convexity of the constraints still ensures some level of solution tractability. In this scenario, it is conceivable that the non‐smooth convex problem can be transformed into a linear programming feasibility problem with equivalence. As a result, RBA problems can be numerically resolved with the same level of efficiency as FBA problems.

#### 2.4.3 Expression and thermodynamics flux models

Expression and thermodynamics flux models (ETFL) is one of the fine‐grained methods and explicitly takes into account the transcriptomics and translational mechanism beside metabolic network. ETFL considers constraints of metabolic processes and how genes are transcribed into messenger RNA and translated into proteins (Salvy & Hatzimanikatis, 2020). Making enzymes requires energy and resources, and this cost needs to be factored into the overall picture of how a cell's metabolism operates. In the case of the ETFL models, the expression cost encompasses several factors. This includes the energy and resources needed to synthesize both peptides and messenger RNA. Additionally, there is consideration for the competition for limited cellular resources such as ribosomes and RNA polymerase.

ETFL tries to bridge biochemistry to computational optimization by driving governing equations and mechanisms for each step. ETFL uses mass balance‐based equation for every macromolecules as follows:

(16) $$v_{i}^{\mathrm{syn}}-v_{i}^{\deg}-\mu \times G_{i}=0,$$

where the $v_{i}^{\mathrm{syn}}$ and $v_{i}^{\deg}$ are rates of synthesis and degradation of macromolecule *i*, respectively. Also, μ is the specific growth rate, $G_{i}$ stands for the dilution of macromolecule i in the cell, and $\mu \times G_{i}$ represents dilution rate of macromolecule *i*. ETFL, like FBA, ignores the dilution rate for metabolites but considers the dilution rate for macromolecules. The reason behind this assumption lies in the differing quantities of macromolecules and metabolite flux values. The closer a reaction is to central carbon metabolism, the higher the flux it would carry. As the reactions associated with macromolecules are distant from central carbon metabolism, they carry comparatively less flux. Therefore, the dilution rate would be considered negligible for metabolites, as it is low compared to metabolite fluxes. On the other hand, it would be significant for macromolecules.

In order to connect metabolites to expression data, ETFL narrows the allowable flux values for each reaction v using enzyme concentration E values and enzyme turnover rates $k_{\mathrm{cat}}$, similar to other methods using Equation (9).

The same procedure is also applied for constraining peptides and mRNA synthesis. Bringing in the peptides, mRNA, enzymes and their corresponding reactions adds an extra space to the stoichiometric matrix termed an expression problem (EP).

Integrating additional constraints in metabolic models may introduce additional mathematical or numerical optimization challenges. In many instances, ME‐models encounter non‐linear growth maximization due to the consideration of macromolecule dilutions. This transformation into non‐linearity becomes evident from Equation (16), as the dilution rate is a product of two variables. To tackle this challenge, specialized solution algorithms have been developed. An example of these methods is solveME (Yang et al., 2016). Alternatively, ETFL discretizes and linearizes the bilinear products, thus transforming the problem into a MILP. To achieve this, ETFL employs a piece‐wise constant function as an approximation to the growth rate. Consequently, the term $\mu \times G_{i}$ becomes a piece‐wise linear function, which aligns well with the requirements of a MILP problem. This discretization approach involves converting the continuous growth rate variable into a sum of products of binary variables. Subsequently, ETFL applies the Petersen linearization method (Petersen, 1971) to further simplify the problem. This involves converting the aforementioned product into a linear system, introducing one new variable and three new constraints. This systematic procedure is extended to all mass‐balance equations concerning macromolecules‐encompassing enzymes, mRNAs, charged tRNAs, and uncharged tRNAs. Consequently, this treatment renders all mass‐balance equations linear. By employing these techniques, the ETFL method utilizes COBRA models as a scaffold for constructing an ETFL model.

The hypothesis behind minimization of metabolite adjustment (MOMA) (Segre et al., 2002) is extended in the formulation of ETFL with minimization of protein adjustment (MOPA), minimization of mRNA adjustment (MORA), and minimization of expression adjustment (MOXA). Mixed integer quadratic programming (MIQP) is required to solve such problems which may be numerically challenging, due to the non‐convexity of the feasible set and the incorporation of integer variables, especially for high dimensional models.

## 3 CHALLENGES

Integrating genome‐scale metabolic models with proteomics data presents various challenges. As an initial step, it is crucial to comprehend these challenges in order to effectively overcome them. In this section, we discuss three of the most significant challenges that every method must address:

### 3.1 In vivo versus in vitro

The regulation of metabolism in living organisms is influenced not only by enzyme kinetics but also by changes in substrate and product concentrations across different physiological states, as well as the response of enzymes to the presence of other metabolites at physiological concentrations. Previous studies have confirmed that incorporating in vivo turnover numbers leads to more predictive models compared to relying solely on in vitro data (Broddrick et al., 2022; Heckmann et al., 2018; Wendering et al., 2022). However, the current challenge lies in the limited availability of in vivo data. Two studies (Cotten & Reed, 2013; Goelzer et al., 2015), have demonstrated the generation of highly accurate results by incorporating in vivo omics data. Additionally, a recent study focused on *Chlamydomonas reinhardtii* derived enzyme turnover numbers from in vivo protein abundance data (Arend et al., 2023). This study highlights that integrating in vivo turnover numbers into a protein‐constrained metabolic model of *C. reinhardtii* improves the accuracy of predicted enzyme usage compared to using in vitro turnover numbers. Another study investigates the stability of in vivo enzyme turnover numbers under various genetic perturbations and experimental conditions in *E. coli* (Heckmann et al., 2020). Using absolute proteomics and fluxomics data, this study finds in vivo turnover numbers to be robust against genetic perturbations. Machine learning models are then trained separately on in vivo and in vitro data to extrapolate reactions in a genome‐scale model. The findings indicate that machine learning and genome‐scale models using in vivo $k_{\mathrm{cat}}$ outperform those using in vitro $k_{\mathrm{cat}}$.

### 3.2 Enzyme turnover number

The turnover number, also known as $k_{\mathrm{cat}}$, represents the highest rate at which a single active site of an enzyme can transform molecular substrates into products. Enzyme turnover numbers play a crucial role in enzyme‐constrained models as they serve as a significant factor linking enzyme and metabolic concentration to metabolic flux reaction values. Several studies have confirmed the strong interdependence between enzyme turnover numbers and the accuracy of model predictions. A common challenge among many methods is the acquisition of enzyme turnover numbers. While there exist a variety of approaches for calculating or retrieving enzyme turnover numbers, this section will focus on the three most frequently used methods. Table 2 summarizes how some of the most common methods determines the enzyme turnover number.

**TABLE 2:** Methods for retrieving and calculating enzyme turnover rates.

Method	Enzyme turnover rate retrieval method	Reference of method	Reference of database (if applicable)
MOMENT	Database (BRENDA & SABIO‐RK)	Adadi et al. (2012)	Chang et al. (2021) and Wittig et al. (2006)
GECKO	Database (BRENDA)	Sánchez et al. (2017)	Chang et al. (2021)
GECKO 2.0	Database (BRENDA)	Domenzain et al. (2022)	Chang et al. (2021)
PAM	Database (BRENDA)	Alter et al. (2021)	Chang et al. (2021)
SIMMER	Data regression NNLS	Hackett et al. (2016)	‐
DLKcat	Deep learning approach	Li et al. (2022)	‐
IOMA	Database (BRENDA)	Yizhak et al. (2010)	Chang et al. (2021)

#### 3.2.1 Databases to retrieve turnover number

Experimental records of enzyme turnover rates are both sparse and subject to noise. However, online databases such as the BRENDA and the SABIO‐RK database provide a wealth of information about enzyme kinetics, including turnover numbers. Most of the methods that integrate genome‐scale models with proteomics use these databases as their main reference for retrieving essential data like enzyme turnover numbers. The BRENDA database represents an extensive compilation of enzyme and metabolic information derived from primary literature sources. It encompasses a diverse range of data, incorporating at least 83,000 distinct enzymes from 9,800 organisms, classified into approximately 4,200 EC numbers (Schomburg et al., 2004). The accessibility of the database is widespread, offering free online access as well as an in‐house version for commercial users. Notably, the BRENDA enzyme database facilitates the visualization and comparison of numerical parameters, enabling users to assess the distribution of functional values. This feature proves invaluable for determining the comparability of measured values and identifying potential outliers. Regular updates to the database introduce new releases that incorporate novel and updated enzyme classes (Chang et al., 2021). SABIO‐RK is a database that provides comprehensive information about biochemical reactions and their kinetic properties. The database was established in 2006 to support researchers and scientists who are interested in studying biochemical reactions and complex networks. The data in SABIO‐RK are curated and annotated by experts in the field and are stored in a relational database. The database is web‐accessible and offers standardized data that are manually extracted from the literature and data directly submitted from lab experiments. SABIO‐RK is an important resource for researchers who are interested in studying biochemical reactions and their kinetics (Wittig et al., 2006). Methods that rely on database retrieval of data may encounter situations where specific data is not available for the microorganism of interest or the particular condition under consideration, for example, the correct pH, temperature or ionic strength. In such cases, these methods typically resort to using mean turnover number values derived from different species or across all reactions in the metabolic model.

#### 3.2.2 Fitting to determine turnover number

One approach to determine enzyme turnover numbers is through data regression. In a significant study, enzyme concentrations is related to flux values using Michaelis–Menten kinetics (Hackett et al., 2016). To obtain non‐negative values for the turnover numbers $\left(k_{\mathrm{cat}}\right)$ and determine their values, the non‐negative least squares method is employed. By considering experimental flux values and enzyme concentrations, this approach aims to identify the most predictive turnover numbers. This method can provide accurate estimations when both flux measurements and proteomics data are available together.

#### 3.2.3 Machine learning approach for determining turnover number

The integration of proteomics data with Genome‐Scale Metabolic Models (GEMs) has received significant enhancement through the application of machine learning techniques. Machine learning algorithms have played a pivotal role in accurately estimating enzyme turnover rates, providing valuable insights into cellular metabolism (Boorla et al., 2022). The notion that an enzyme's effective turnover rate $k_{\mathrm{eff}}$ tends to be lower than in vitro $k_{\mathrm{cat}}$ due to factors like incomplete saturation, thermodynamic effects, post‐translational modifications, and allosteric regulation was introduced in 2018 (Heckmann et al., 2018). Given the scarcity of in vitro data for both $k_{\mathrm{eff}}$ and $k_{\mathrm{cat}}$, a machine learning model was developed to reveal the underlying relationships among enzyme catalytic turnover rate, enzyme biochemistry, protein structure, and network context. The study uses the linear elastic net, the decision‐tree‐based random forest model, and the complex neural network as three types of machine learning algorithms to construct three different ML models. Utilizing MOMENT (Adadi et al., 2012) and iJL1678b ME‐model (Lloyd et al., 2018; O'Brien et al., 2013), This investigation assessed whether turnover rates predicted by the ML model could enhance predictions of genome‐scale metabolic models. The study showed that ML model‐predicted turnover rates yielded more accurate predictions in both MOMENT and ME‐model compared to predictions incorporating in vitro $k_{\mathrm{cat}}$.

One recent study introduces DLKcat, a machine learning framework designed to predict $k_{\mathrm{cat}}$ values for metabolic enzymes across a broad spectrum of organisms (Li et al., 2022). DLKcat adopts a representation‐based learning paradigm wherein enzyme amino acid sequences and substrate structures are discretized into sequence words and substrate substructures, respectively. Employing convolutional neural networks and graph neural networks, salient features are captured from these representations. Subsequently, a fully connected neural network layer predicts the enzyme turnover numbers. By extensively training on substantial datasets sourced from prominent databases such as BRENDA and SABIO‐RK, DLKcat achieves commendable accuracy, substantiated by a high correlation coefficient.

A significant study introduced TurNup, an organism‐independent model capable of predicting in vitro enzyme turnover numbers for natural reactions of wild‐type enzymes (Kroll et al., 2023). One key advantage of TurNup over previously published models is its incorporation of comprehensive chemical reaction information as input. This is achieved by taking into account numerical fingerprints that encompass the complete set of substrates and products involved in a reaction. Furthermore, TurNup includes enzyme characteristics as part of its input data. Unlike previous studies that utilized convolutional neural networks, TurNup employs a transformer language model, which is a deep neural network tailored for sequence processing to represent enzyme properties (Rives et al., 2021). Consequently, TurNup surpasses other methods due to its incorporation of additional inputs and the utilization of more advanced models for enzyme sequence processing.

### 3.3 Trade‐off: model accuracy, computational cost, and data scarcity

The third challenge discussed here pertains to the trade‐off between model accuracy, computational cost, and data scarcity. Fine‐grained approaches offer more experimentally consistent results but require a larger amount of biological and kinetic data, which may not be feasible to obtain for all species. Conversely, simpler approaches require less biological data but are generally associated with lower accuracy. In terms of computational considerations, approaches incorporating more complex mechanisms often involve more challenging problem types, such as mixed‐integer quadratic programming (MIQP). In contrast, simpler methods can be implemented using less computationally demanding techniques, such as quadratic programming (QP), linear programming (LP), and convex optimization.

## 4 CONCLUSIONS

The integration of proteomics with COBRA models has shown promising results in generating predictive models with increased accuracy. The future is likely to witness a growing number of enzyme‐constrained genome‐scale models, inspired by the considerable attention given to pioneering methods (Beg et al., 2007; Sánchez et al., 2017). Conversely, while ME‐models have demonstrated increased accuracy, their applications have remained limited to relatively fewer case studies, perhaps due to their complexity and the challenges associated with manually gathering extensive biochemical information. In situations where abundant biological data and advanced computational systems are available, fine‐grained approaches are considered ideal. However, when faced with limited data availability and less powerful computing resources, simpler approaches may be preferable.

Overcoming the discussed challenges and striving to enhance the methods employed will lead to the development of more powerful models. For the retrieval of enzyme turnover numbers, accessing enzymatic constants from databases emerges as a rational solution, as many methods have adopted this approach. It is worth noting that the use of databases can yield more precise results when substituting missing data for an enzyme by selecting data based on similarity to the original organism and the desired phenotype, rather than relying on random selection or mean values across various organisms, as demonstrated in GECKO 2.0 (Domenzain et al., 2022). However, with the emergence of powerful machine learning algorithms, it becomes more reasonable to employ machine learning approaches instead of relying solely on in vitro data retrieval from databases or fitting methods, as they tend to yield more accurate results, particularly when dealing with large datasets. Moreover, utilizing in vivo omics data holds greater promise compared to in vitro data. However, in many instances, in vivo data is not readily accessible, leading researchers to resort to using in vitro data. Nonetheless, exploring the possibility of generating kinetic constants, such as enzyme turnover numbers, using machine learning methods can be an innovative approach. By leveraging the limited available in vivo omics data as training sets, it may be possible to generate more accurate models that are based on in vivo conditions (Arend et al., 2023; Heckmann et al., 2018, 2020).

## AUTHOR CONTRIBUTIONS

**Farid Zare:** Writing – original draft; writing – review and editing; investigation. **Ronan M. T. Fleming:** Writing – review and editing; validation; project administration; supervision; funding acquisition.
